# A nomogram constructed using intraoperative ex vivo shear-wave elastography precisely predicts metastasis of sentinel lymph nodes in breast cancer

**DOI:** 10.1007/s00330-019-06473-5

**Published:** 2019-11-06

**Authors:** Soong June Bae, Ji Hyun Youk, Chang Ik Yoon, Soeun Park, Chi Hwan Cha, Hak Woo Lee, Sung Gwe Ahn, Seung Ah Lee, Eun Ju Son, Joon Jeong

**Affiliations:** 1 Department of Surgery, Gangnam Severance Hospital, Yonsei University College of Medicine, 211 Eonju-ro, Gangnam-gu, Seoul, 06273 Republic of Korea; 2 Department of Radiology, Gangnam Severance Hospital, Yonsei University College of Medicine, Seoul, Republic of Korea; 3 Department of Surgery, St Mary’s Hospital, College of Medicine, The Catholic University of Korea, Seoul, Republic of Korea; 4 Department of Surgery, CHA Bundang Medical Center, CHA University, Seongnam, Republic of Korea

**Keywords:** Breast neoplasm, Elasticity imaging techniques, Nomogram, Lymphatic metastasis

## Abstract

**Objective:**

To develop a nomogram and validate its use for the intraoperative evaluation of nodal metastasis using shear-wave elastography (SWE) elasticity values and nodal size

**Methods:**

We constructed a nomogram to predict metastasis using ex vivo SWE values and ultrasound features of 228 axillary LNs from fifty-five patients. We validated its use in an independent cohort comprising 80 patients. In the validation cohort, a total of 217 sentinel LNs were included.

**Results:**

We developed the nomogram using the nodal size and elasticity values of the development cohort to predict LN metastasis; the area under the curve (AUC) was 0.856 (95% confidence interval (CI), 0.783–0.929). In the validation cohort, 15 (7%) LNs were metastatic, and 202 (93%) were non-metastatic. The mean stiffness (23.54 and 10.41 kPa, *p* = 0.005) and elasticity ratio (3.24 and 1.49, *p* = 0.028) were significantly higher in the metastatic LNs than those in the non-metastatic LNs. However, the mean size of the metastatic LNs was not significantly larger than that of the non-metastatic LNs (8.70 mm vs 7.20 mm, respectively; *p* = 0.123). The AUC was 0.791 (95% CI, 0.668–0.915) in the validation cohort, and the calibration plots of the nomogram showed good agreement.

**Conclusions:**

We developed a well-validated nomogram to predict LN metastasis. This nomogram, mainly based on ex vivo SWE values, can help evaluate nodal metastasis during surgery.

**Key Points:**

*• A nomogram was developed based on axillary LN size and ex vivo SWE values such as mean stiffness and elasticity ratio to easily predict axillary LN metastasis during breast cancer surgery.*

*• The constructed nomogram presented high predictive performance of sentinel LN metastasis with an independent cohort.*

*• This nomogram can reduce unnecessary intraoperative frozen section which increases the surgical time and costs in breast cancer patients.*

## Introduction

Axillary lymph node (LN) metastasis is one of the important prognostic factors in breast cancer [1, 2]. Sentinel LN biopsy (SLNB) has become the standard method for axillary staging in patients with clinically negative nodes [3]. Further, intraoperative pathologic assessment of sentinel LNs enables surgical staging and aids in surgical decision-making regarding whether axillary LN dissection (ALND) should be performed during surgery [4–7]. However, it requires skilled pathologists and equipment, which increases the surgical time and costs.

Shear-wave elastography (SWE) can quantitatively calculate the elasticity parameters of target lesions and provide values, such as mean, minimum, and maximum stiffness; standard deviation of elasticity; and elasticity ratio of breast lesions to the adjacent fat tissue. It is helpful in distinguishing breast cancerous lesions because malignant tissues tend to have increased stiffness compared with benign tissues [8–12]. Also, SWE well predicted the histological upgrade to invasive cancer in ductal carcinoma in situ confirmed at biopsy and response to neoadjuvant chemotherapy in breast cancer [13, 14]. Furthermore, several studies reported that SWE could be applied to identify axillary LN metastasis as well as breast malignancy [15–19]. In addition, we previously reported that the nodal size and elasticity values, such as maximum stiffness, mean stiffness, and elasticity ratio of axillary LNs to the adjacent fat tissues, were associated with metastatic axillary LNs [20]. Therefore, ex vivo SWE is thought to be a feasible method to predict axillary LN metastasis.

Based on these results, we hypothesized that the nomogram constructed using ultrasound features and elasticity values measured via intraoperative ex vivo SWE can accurately predict nodal metastasis. Furthermore, the disadvantages of intraoperative frozen section analysis may be compensated if the nomogram precisely predicts sentinel LN metastasis, since ex vivo SWE can be performed easily, and no additional equipment is required, except for ultrasound. For this reason, we developed and validated a nomogram to predict the sentinel LN metastasis using ultrasound features and ex vivo SWE values in this study.

## Materials and methods

### Patients

In our previous prospective study, a total of 228 axillary LNs, including sentinel LNs obtained from fifty-five patients who underwent breast cancer surgery in Gangnam Severance Hospital from May 2014 to April 2015, were investigated [20]; these patients were utilized as the development cohort. Another 80 patients diagnosed with breast cancer underwent breast cancer surgery at the same hospital from August 2015 to March 2016 were prospectively enrolled. Patients with stage IV cancer or those who received neoadjuvant chemotherapy were excluded. Unlike in the development cohort, 217 sentinel LNs obtained from these 80 patients examined using intraoperative ex vivo SWE were included in the validation cohort.

Our study was approved by the Institutional Review Board of Gangnam Severance Hospital and conducted in accordance with the Good Clinical Practice guidelines and the Declaration of Helsinki principles.

### SLNB

SLNB was performed using 99mTc-labeled tin colloid. Intradermal injection of 0.4 mL 30 MBq (0.8 mCi) 99mTc tin colloid diluted in normal saline solution was performed at four peri-areolar sites. Sentinel LNs were determined by employing a gamma detector (Gamma Detection System, Neoprobe Corporation). The LNs showing a high radioactivity were dissected, after which the gamma detector was used again to confirm the correct sentinel LNs. All radioactive LNs with an account equal to or greater than 10% of the highest radioactive LN were removed. When suspicious LNs not detected by the gamma probe were found after SLNB, they were removed and examined as non-sentinel LNs. If the result of frozen pathology was positive for malignancy, ALND was performed.

### B-Mode ultrasound with ex vivo SWE

**Fig. 1:**
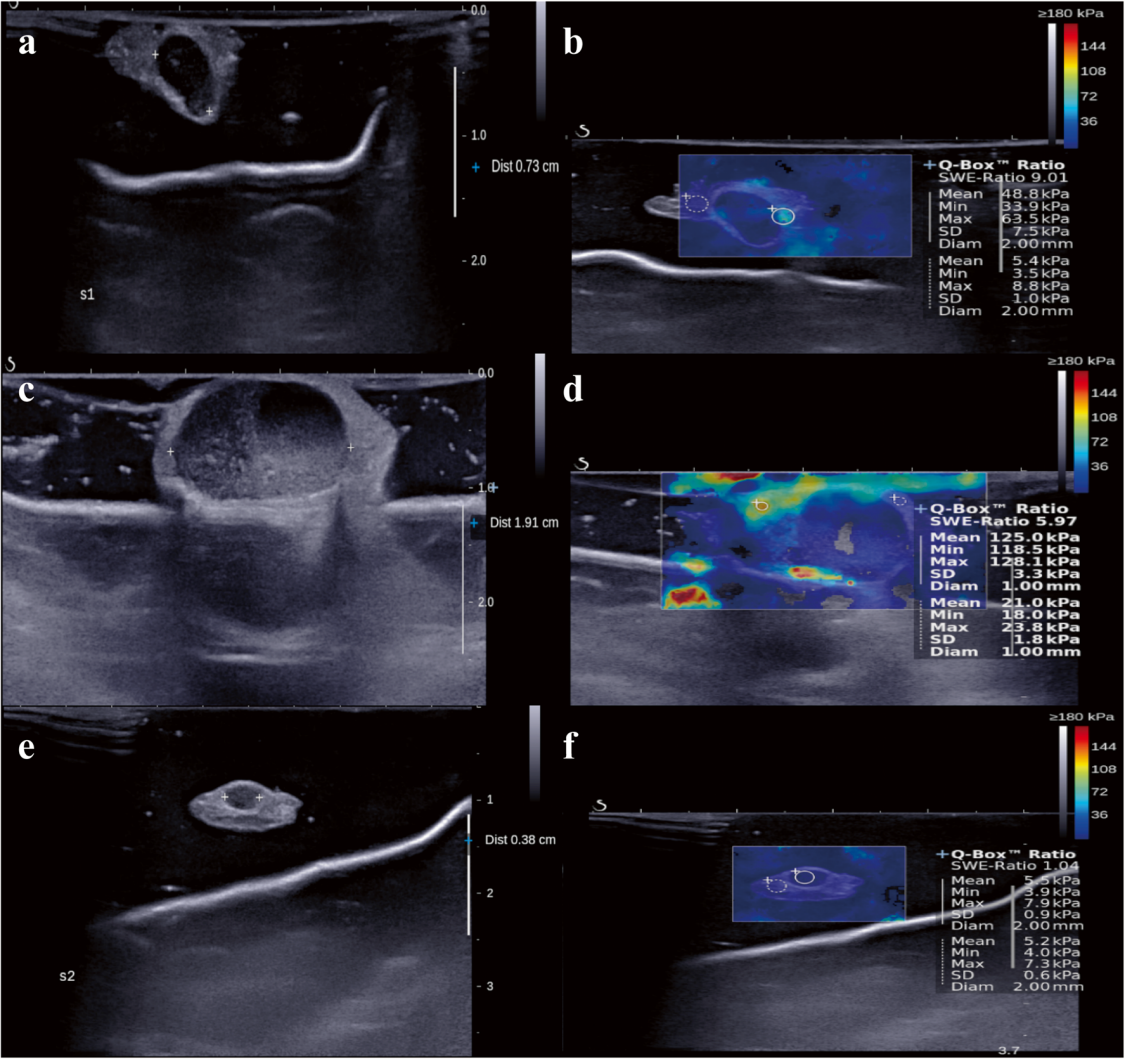
B-Mode ultrasound and shear-wave elastography (SWE). **a** B-Mode ultrasound showed 0.73-cm–sized excised sentinel lymph node with adjacent fat tissue. **b** The mean stiffness was measured by placing the 2-mm–sized region-of-interest (ROI) on the stiffest part of the excised sentinel lymph node (circle). The stiffness of the adjacent fat tissue was measured by placing another ROI on the surrounding fat tissue (dotted circle). Then, the elasticity ratio was calculated automatically. **a**–**d** B-Mode ultrasound and shear-wave elastography (SWE) images of patients with lymph node metastasis. **e**, **f** B-Mode ultrasound and shear-wave elastography (SWE) images of patient without lymph node metastasis

Excised LNs were delivered to the radiology part. Then, B-mode ultrasound with ex vivo SWE was performed as mentioned in our previous study [20]. In summary, the nodal size and elasticity values, such as mean stiffness and elasticity ratio of the sentinel LNs to the adjacent fat tissues, were examined during surgery via B-mode ultrasound and SWE using the Aixplorer ultrasound system (Super Sonic Imagine) and ShearWave^TM^ elastography mode [21, 22] with a 4–15-MHz linear transducer. During the examination, the radiologists need to handle the specimen carefully so as not to exert pressure on the excised LNs by the ultrasound transducer and sufficient contact jelly was used to avoid artificial stiffness. The longest diameter of the LN which was hypoechoic compared with the adjacent fat tissue was measured in B-mode ultrasound (Fig. 1). After few seconds of immobilization to allow the SWE image to stabilize, mean stiffness was measured by placing a fixed 2-mm circular region-of-interest (ROI) box (Q-BOX^TM^; SuperSonic Imagine) on the stiffest region of the excised LNs. The elasticity ratio was calculated automatically when the first ROI was placed on the stiffest region of excised LNs, and the other ROI was immediately placed on the adjacent fat tissue (Fig. 1). Four radiologists with a clinical experience of more than 2 years performed B-mode ultrasound with SWE, and one radiologist with more than 10 years of clinical experience reviewed the results, i.e., nodal size and elasticity values, such as mean stiffness and elasticity ratio.

### Pathologic evaluation of excised LNs

After the radiologic examination was finished, the pathologic evaluation of sentinel LNs was performed. The excised sentinel LNs were cut into 2–3-mm sizes, and all slices were assessed. The size of the metastatic lesions within the excised nodes was reported in accordance with the staging system of the American Joint Committee on Cancer as follows: > 2 mm, macro-metastasis; 0.2–2.0 mm, micro-metastasis; and < 0.2 mm, isolated tumor cells [23]. In this study, sentinel LN metastasis was defined as macro-metastasis.

### Development of the nomogram and statistical analysis

A nomogram was established on the basis of the results of the multivariate analysis in the development cohort. Thereafter, 200 bootstrap samples were used for internal validation of the development cohort, and external validation was performed with an independent cohort. The performance of the constructed nomograms was quantified with respect to discrimination and calibration [24]. The discriminative power of whether the constructed nomogram can correctly predict the probability of LN metastasis was quantified using the area under the receiver operating characteristic curve. Calibration was performed to identify the agreement between the observed outcomes and predicted probabilities of metastasis among the excised LNs. To verify the suitability of the constructed nomograms, the Hosmer-Lemeshow goodness-of-fit test was performed [25, 26]. The software used to perform these analyses was the SAS program (ver 9.2, SAS Institute Inc.) or the R Statistical Package (ver 3.3.3, Institute for Statistics and Mathematics).

The characteristics of the patients were analyzed using the chi-square test and independent two-sample *t* test. The mean elasticity values were compared between the non-metastatic and metastatic sentinel LNs using the independent two-sample *t* test. Analysis was performed using the SPSS software ver 23 (SPSS).

## Results

### Nomogram construction using the development cohort and internal validation

**Fig. 2:**
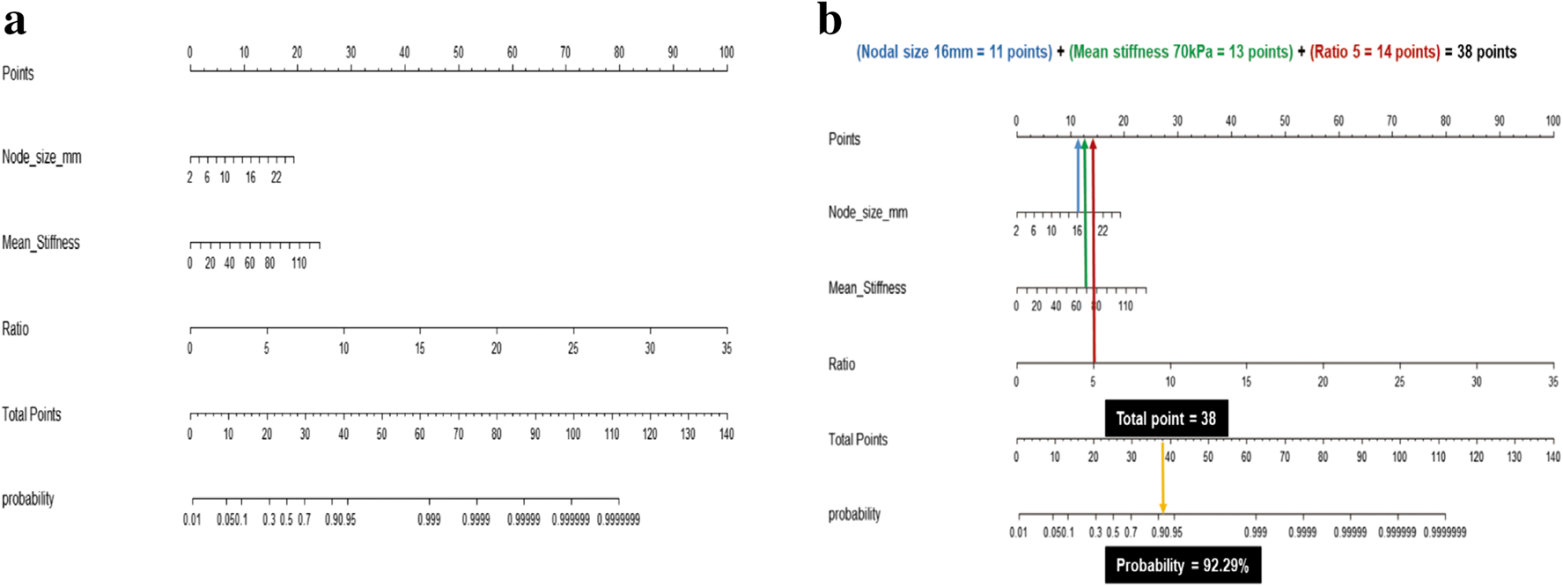
Constructed nomogram. **a** Constructed nomogram based on the predictive factors of axillary LN metastasis, such as nodal size, mean stiffness, and elasticity ratio. **b** Example of constructed nomogram. LN, lymph node

We previously reported the characteristics and elasticity values of the harvested axillary LNs measured using B-mode ultrasound and intraoperative ex vivo SWE in the development cohort [20]. Briefly, the nodal size and elasticity values, such as mean stiffness and elasticity ratio, were associated with axillary LN metastasis in the multivariable analysis. The nomogram was constructed on the basis of significant factors from the multivariable analysis in the development cohort to predict the probability of axillary LN metastasis (Fig. 2a). From binary logistic regression model for multivariable analysis, we obtained intercept and regression coefficient of each significant factor. The probability of axillary LN metastasis was calculated as follows: 1/(1 + exp(−A)), where *A* =  − 4.9898 + 0.1498 × (nodal size) + 0.0345 × (mean stiffness) + 0.5321 × (ratio)

In the nomogram, the estimated probability of LN metastasis could be obtained by summing the scores of each variable and locating such on the total score scale. For instance, a patient with a 16-mm nodal size (11 points), 70-kPa mean stiffness (13 points), and 5 ratio (14 points) would score 38 points, which can be converted into 92.29% probability of LN metastasis (Fig. 2b).

**Fig. 3:**
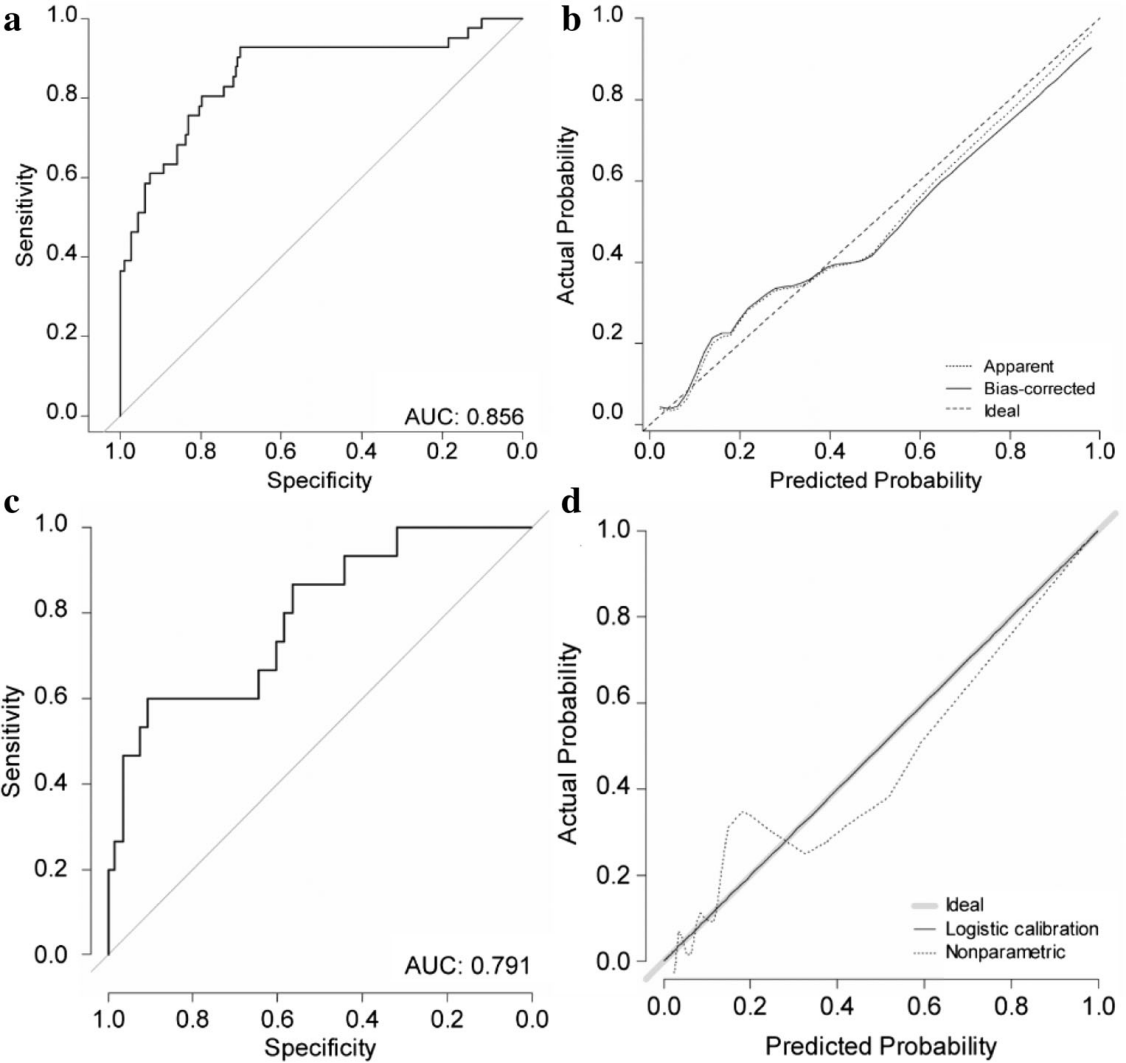
ROC curve of the nomogram and the calibration plot. **a** ROC curve of the nomogram; **b** the calibration plot in the development cohort with internal validation; **c** ROC curve of the nomogram; **d** the calibration plot in the validation cohort with external validation. ROC, receiver operating characteristic

The area under the curve (AUC) was 0.856 (95% confidence interval (CI), 0.783–0.929) in the development cohort (Fig. 3a). The *p* value obtained using the Hosmer-Lemeshow goodness-of-fit test was 0.354, indicating a good fit of the model. When internal validation was performed using the 200 bootstrap samples, the mean absolute error was as low as 0.029. Moreover, the calibration plots of the nomogram showed good agreement between the observed and predicted outcomes (Fig. 3b).

### Validation cohort

In the validation cohort, 10 of the 80 patients had sentinel LN metastases, all of which were in the N1 stage. Tumor size of the patients with sentinel LN metastasis was larger than that of the patients without sentinel LN metastasis (2.35 cm vs 1.61 cm, *p* = 0.045). The other clinicopathologic factors were no significantly different between the patients with and without sentinel LN metastasis (Table 1). The median age of all patients was 55 (range, 22–76) years. The findings for estrogen receptor (ER) and human epidermal growth factor receptor 2 (HER2) were positive in 67 (83.8%) and 15 (18.8%) patients, respectively. T1 stage and grade 2 were the most common classifications in 52 (65.0%) and 43 (53.8%) patients, respectively. The number of elderly patients and patients with HER2-negative findings and low N stage was higher in the validation cohort than in the development cohort (Table 2).

**Table 1:** Clinicopathologic characteristics of the validation cohort according to LN metastasis

Variables	Patients with non-malignant sentinel LN (*N* = 70)	Patients with malignant sentinel LN (*N* = 10)	All patients (*N* = 80)	*p* value
Median age (years)	55.5 (35–76)	54 (22–75)	55 (22–76)	0.716
Tumor size (cm)	1.61 ± 1.07	2.35 ± 1.14	1.68 ± 1.06	0.045
T stage				0.520^†^
T1mi	2 (2.9%)	0	2 (2.5%)	
T1	47 (67.1%)	5 (50.0%)	52 (65.0%)	
T2	20 (28.6%)	5 (50.0%)	25 (31.3%)	
T3	1 (1.4%)	0	1 (1.2%)	
HG				0.312^†^
Grade I	18 (25.7%)	1 (10.0%)	19 (23.8%)	
Grade II	38 (54.3%)	5 (50.0%)	43 (53.8%)	
Grade III	14 (20.0%)	4 (40.0%)	18 (22.5%)	
ER				0.662^†^
Positive	59 (84.3%)	8 (80.0%)	67 (83.8%)	
Negative	11 (15.7%)	2 (20.0%)	13 (16.2%)	
PR				0.715^†^
Positive	48 (68.6%)	8 (80.0%)	56 (70.0%)	
Negative	22 (31.4%)	2 (20.0%)	24 (30.0%)	
HER2				0.086^†^
Positive	11 (15.7%)	4 (40%)	15 (18.8%)	
Negative	59 (84.3%)	6 (60%)	65 (81.3%)	

*LN*, lymph node; *HG*, histologic grade; *ER*, estrogen receptor; *PR*, progesterone receptor; *HER2*, human epidermal growth factor receptor 2

^†^Fisher’s exact test

**Table 2:** Comparison of the clinicopathologic characteristics between the development and validation cohorts

Characteristics	Development cohort (*N* = 55)	Validation cohort (*N* = 80)	*p* value
Median age, years (range)	49 (31–69)	55 (22–76)	0.009*
Tumor size, cm (range)	1.75 (0.10–3.60)	1.70 (0.1–6.20)	0.766
T stage			0.382^†^
Tis	2 (3.6%)	0	
T1mi	2 (3.6%)	2 (2.5%)	
T1	31 (56.4%)	52 (65.0%)	
T2	20 (36.4%)	25 (31.3%)	
T3	0	1 (1.3%)	
N stage			< 0.001^†^
0	28 (51.0%)	70 (87.5%)	
N1	19 (34.5%)	10 (12.5%)	
N2	6 (11.0%)	0	
N3	2 (3.5%)	0	
HG^a^			0.096
Grade I	7 (12.7%)	19 (23.8%)	
Grade II	25 (45.5%)	43 (53.8%)	
Grade III	20 (36.4%)	18 (22.5%)	
ER			0.074
Positive	39 (70.9%)	67 (83.8%)	
Negative	16 (29.1%)	13 (16.2%)	
PR			0.157
Positive	32 (58.2%)	56 (70.0%)	
Negative	23 (41.8%)	24 (30.0%)	
HER2			0.012
Positive	21 (38.2%)	15 (18.8%)	
Negative	34 (61.8%)	65 (81.3%)	

*HG*, histologic grade; *ER*, estrogen receptor; *PR*, progesterone receptor; *HER2*, human epidermal growth factor receptor 2

*Mann-Whitney test

^†^Fisher’s exact test

^a^Missing data

**Fig. 4:**
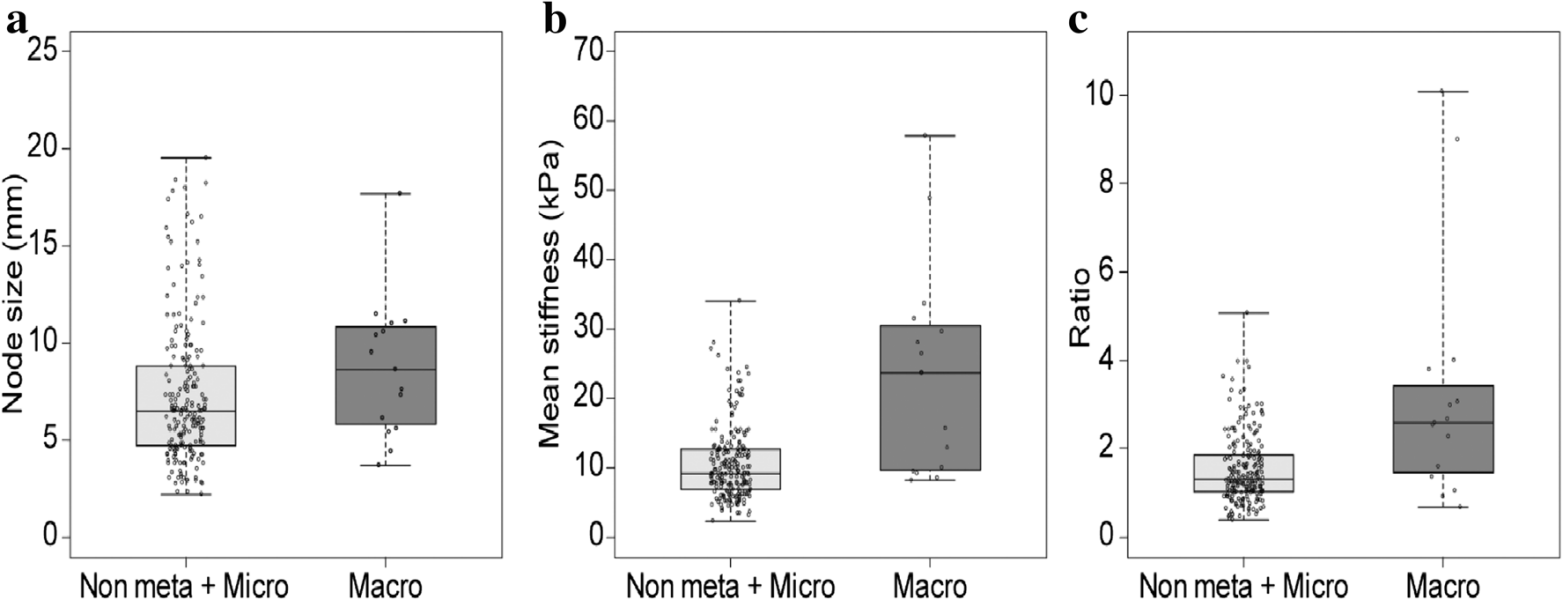
Ultrasound feature and elasticity values between non-metastatic and metastatic lymph nodes. Box plot of the (**a**) nodal size, (**b**) mean stiffness, and (**c**) elasticity ratio between the non-metastatic and metastatic lymph nodes

Of the 217 sentinel LNs, 15 (6.9%) were metastatic, and 202 (93.1%) were non-metastatic, including seven (3.2%) micro-metastatic LNs. The metastatic LNs tended to be larger than non-metastatic LNs, but it was not significant (8.70 mm vs 7.20 mm, *p* = 0.123) (Table 3). The mean stiffness of the metastatic sentinel LNs was significantly greater than that of the non-metastatic sentinel LNs (23.54 vs 10.41 kPa, *p* = 0.005) (Fig. 4). Furthermore, the elasticity ratio of the metastatic sentinel LNs was significantly higher than that of the non-metastatic sentinel LNs (3.24 vs 1.49, *p* = 0.028). Meanwhile, there was no difference in nodal size and elasticity values according to histological types of breast cancer (Table 4).

**Table 3:** Ultrasound features and elasticity values measured using intraoperative ex vivo shear-wave elastography and total points in the nomogram between the non-metastatic and metastatic axillary LNs

	Total (*n* = 217)	Non-metastatic sentinel LNs (*n* = 202)	Metastatic sentinel LNs (*n* = 15)	*p* value
Nodal size (mm)	7.31 ± 3.63	7.20 ± 3.62	8.70 ± 3.60	0.123
Mean stiffness (kPa)	11.32 ± 7.2	10.41 ± 5.24	23.54 ± 15.28	0.005
Elasticity ratio	1.61 ± 1.12	1.49 ± 0.77	3.24 ± 2.76	0.028
Total points in nomogram	9.08 (95% CI, 7.54–10.62)	7.67 (95% CI, 6.69–8.64)	28.06 (95% CI, 11.29–44.83)	< 0.001*

*LN*, lymph node

*Mann-Whitney test

**Table 4:** Ultrasound features and elasticity values measured using intraoperative ex vivo shear-wave elastography according to histological types of breast cancer

	Histologic type			*p* value
	IDC (*n* = 179)	ILC (*n* = 12)	Others* (*n* = 26)	
Nodal size (mm)	7.13 ± 3.35	7.43 ± 4.99	8.45 ± 4.61	0.225
Mean stiffness (kPa)	11.53 ± 7.56	8.44 ± 2.67	11.15 ± 5.82	0.353
Elasticity ratio	1.60 ± 1.18	1.66 ± 0.64	1.66 ± 0.75	0.960

*IDC*, invasive ductal carcinoma; *ILC*, invasive lobular carcinoma

*Others; medullary carcinoma (*n* = 3), mucinous carcinoma (*n* = 11), tubular carcinoma (*n* = 4), cribriform carcinoma (*n* = 5), apocrine carcinoma (*n* = 3)

### External validation of the nomogram

The nomogram was validated with the independent set including only 217 sentinel LNs. The discriminative power was good, with an AUC of 0.791 (95% CI, 0.668–0.915) (Fig. 3c). The calibration plots of the nomogram showed good agreement between the observed and predicted outcomes (Fig. 3d).

## Discussion

In this study, we generated a nomogram to predict metastatic LNs based on nodal size and elasticity values such as mean stiffness and elasticity ratio of harvested LNs on B-mode ultrasound with ex vivo SWE using data from our previous study. Thereafter, we tested the predictive ability of the nomogram in an independent set consisting of sentinel LNs. The performance of constructed nomogram to predict sentinel LNs metastasis was high in terms of good discrimination and calibration.

Cancer researchers, clinicians, and the public are becoming increasingly interested in statistical models designed to predict the occurrence or the outcome of cancer, along with the efficacy of treatments [25–27]. Among several prediction models, nomograms have been shown to provide personalized reasonable risk estimates that facilitate management-related decisions [27]. Indeed, our nomogram can be used to calculate the probability of each axillary LN metastasis easily and rapidly. When the total point calculated using the nodal size, mean stiffness, and elasticity ratio is over 35 points, the probability of LN metastasis exceeds 90%. If the total point is over 48 points, the probability of LN metastasis exceeds 99%. Indeed, the mean total point in the nomogram was significantly different between the non-metastatic and metastatic sentinel LNs (Supplementary Table 3). In the metastatic sentinel LNs, the probability of LN metastasis was approximately 70%, as the mean total point was 28 points; in the non-metastatic sentinel LNs, the probability of LN metastasis was less than 10%, as the mean total point was 8 points.

The discriminative power of the nomogram was quantified using the AUC exhibiting the accuracy of the test, with an AUC of 0.5 being defined as non-informative; 0.5–0.7, fair; 0.7–0.9, good; and > 0.9, excellent. Our nomogram revealed good discriminative power with the AUC of 0.856 in the development cohort and 0.791 in the validation cohort. In addition, the calibration plot showed good agreement between the observed and predicted probabilities in both the development and validation cohorts. Therefore, our constructed nomogram was suitable in predicting the probability of metastasis among the harvested sentinel LNs.

In the validation cohort, the ultrasound and SWE characteristics of sentinel LNs were not different between histologic types of breast cancer in line with previous report [28]. The mean stiffness and elasticity ratio of the metastatic sentinel LNs were significantly higher than those of the non-metastatic LNs. These findings are similar to those of the development cohort in our previous study. The nodal size tended to be larger in the metastatic LNs than in the non-metastatic LNs, although the trend was not significant. It was presumed that the proportion of the metastatic LNs was higher in the development cohort than in the validation cohort (Table 2). Further research with a larger number of axillary LNs is needed to investigate the accurate relationship between the nodal size and LN metastasis.

Recently, the American College of Surgeons Oncology Group Z0011 prospective, randomized clinical trial changed the standard approach to axillary surgery, showing that the omission of ALND was possible in early breast cancer patients who underwent breast conserving surgery and adjuvant systemic therapy even with 1–2 metastatic sentinel LNs [29, 30]. Several previous trials also reported consistent findings with those of the Z0011 trial [2, 31]. Since these trials presented that ALND could be omitted even in patients with positive sentinel LNs, the need for intraoperative frozen section analysis of sentinel LNs is questionable. In fact, not only ALND but also intraoperative frozen section analysis of sentinel LNs in patients who underwent breast conservative surgery was declined after the Z0011 trial was published [32, 33]. Furthermore, Noordaa et al suggested that omitting intraoperative pathologic assessment of LNs was a reasonable option in patients with a low nodal burden, such as clinically node-negative breast cancer, who were treated with upfront surgery [34].

However, one of the major disadvantages of omitting intraoperative frozen section analysis is secondary surgery for ALND. To date, the exact rate of secondary surgery for ALND after skipping intraoperative frozen section analysis has not been well researched. Nevertheless, concerns of secondary operation for ALND can be reduced if intraoperative pathologic examination was performed for sentinel LNs in suspicion of metastasis, as identified by our nomogram.

Our study has some limitations. First, the cutoff value of nomogram to determine the intraoperative pathologic examination was not clarified in this analysis. Further prospective studies are needed to verify this issue. Second, the rate of metastatic LNs in the validation cohort was lower than that in the development cohort. It was probably because only sentinel LNs were included in the validation set. The number of metastatic LNs was 15 in the validation cohort of our study; nevertheless, these events were statistically sufficient to perform calibration and discrimination to warrant the use of our nomogram. Finally, concerns about the reproducibility of SWE still remained because interobserver or intraobserver agreements for the elasticity values were not assessed in this study. However, the other previous study showed reliable intraobserver and interobserver reproducibilities of SWE [35, 36]. In addition, the reproducibility of SWE was expected to be higher in our study because specialized radiologists examined the LNs by ex vivo SWE that was less likely to interfere with surrounding tissue compared with in vivo SWE. Hence, it might be expected to precisely perform intraoperative ex vivo SWE of excised LNs within a short time.

In conclusion, our well-validated nomogram can be applied to predict nodal metastasis during SLNB. The clinical application of nomogram, which is mainly based on ex vivo SWE values, may help in reducing unnecessary intraoperative frozen section analysis as well as secondary operation rate in breast cancer patients who underwent SLNB.

## Abbreviations

ALND: Axillary lymph node dissection
AUC: Area under the curve
ER: Estrogen receptor
HER2: Human epidermal growth factor receptor 2
HG: Histologic grade
LN: Lymph node
ROI: Region of interest
SLNB: Sentinel lymph node biopsy
SWE: Shear-wave elastography
